# Neural Mechanisms of Cardioprotection

**DOI:** 10.1152/physiol.00037.2013

**Published:** 2014-03

**Authors:** Andrey Gourine, Alexander V. Gourine

**Affiliations:** ^1^Department of Cardiology, Karolinska Institutet, Karolinska University Hospital, Stockholm, Sweden; and; ^2^Neuroscience, Physiology & Pharmacology, University College London, London, United Kingdom

## Abstract

This review highlights the importance of neural mechanisms capable of protecting the heart against lethal ischemia/reperfusion injury. Increased parasympathetic (vagal) activity limits myocardial infarction, and recent data suggest that activation of autonomic reflex pathways contributes to powerful innate mechanisms of cardioprotection underlying the remote ischemic conditioning phenomena.

Ischemic heart disease remains the most common cause of morbidity and mortality in Western societies, although the relative mortality attributable to it has decreased significantly in the last decade. The heart is highly vulnerable to oxygen deprivation, and occlusion of a major coronary artery is followed by myocardial metabolic and functional changes that develop rapidly after cessation of the blood flow. Restitution of the blood supply to an ischemic area is crucial for tissue survival; however, it also results in a cascade of harmful events that lead to what is known as the myocardial reperfusion injury. Its manifestations include arrhythmias, contractile dysfunction, endothelial dysfunction, and lethal reperfusion damage of cardiomyocytes. Since the size of the myocardial infarction is the major determinant of the subsequent development of the congestive heart failure, novel therapeutic treatments are focusing on limiting the extent of myocardial ischemia/reperfusion injury. Animal experimentations demonstrated that the reduction of infarct size can be achieved either pharmacologically or via the recruitment of powerful innate mechanisms, which are highly effective in protecting myocardial tissue against lethal ischemia/reperfusion injury and include ischemic and remote ischemic pre- and postconditioning. Although none of the identified pharmacological strategies have been successfully translated to the clinical setting, promising results of recent trials in patients with acute myocardial infarction (4) give hope that application of the remote ischemic conditioning procedure(s) may become a standard of care. In this review, we focus on these endogenous mechanisms of cardioprotection and discuss recent evidence suggesting that these phenomena are mediated (at least in part) via the recruitment of neural reflex mechanisms.

## Neural Control of the Heart

Cardiac performance is controlled by the parasympathetic (inhibitory) and sympathetic (facilitatory) limbs of the autonomic nervous system. Sympathetic nerves innervate the sinoatrial and atrioventicular nodes, the atria, the ventricles, and the conducting tissue. Parasympathetic (vagal) efferent nerves are well known to control nodal tissues and atria, whereas the role of the parasympathetic innervation of the ventricles remains controversial. The majority of physiology textbooks teach that the vagal innervation of the ventricular system is sparse and that parasympathetic control of the ventricular contractility is insignificant. This view persists in both the scientific and educational literature, despite significant evidence obtained in various species (from rat to man) demonstrating the presence of choline acetyltransferase-positive nerve fibers, acetylcholinesterase enzyme, and muscarinic receptors in both the right and the left ventricles (recently reviewed in Ref. 8). Functional data clearly demonstrate that increased activity of the vagus nerve decreases the force of ventricular contraction independent of its effect on heart rate [see, for example, the papers by Xenopoulos and Applegate (79) and Lewis and colleagues (41) describing significant negative inotropic effect of the vagus nerve stimulation in the experiments conducted in dogs, pigs, and humans].

**FIGURE 1.**
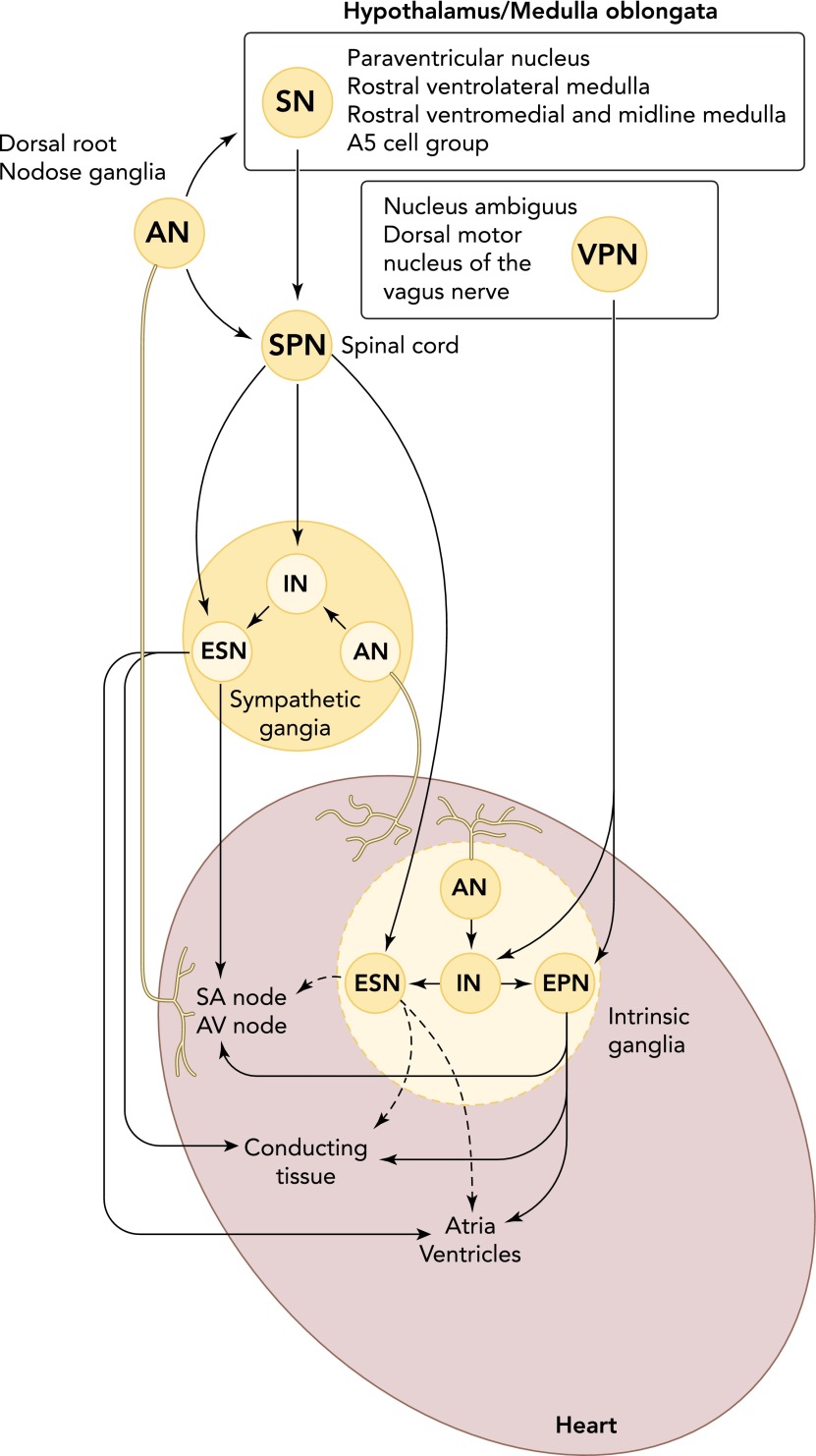
Diagrammatic representation of sensory and efferent neural pathways controlling the heart AN, afferent (sensory) neurones; EPN, efferent parasympathetic neurones; ESN, efferent sympathetic neurones; IN, interneurones; SN, sympathoexcitatory neurones; SPN, sympathetic preganglionic neurones; VPN, vagal (parasympathetic) preganglionic neurones.

The parasympathetic preganglionic neurones that innervate the heart are located in the medulla oblongata, and their axons travel in the vagus nerve to synapse on the parasympathetic efferent postganglionic neurones of the intrinsic cardiac ganglia (FIGURE 1) (30, 72). Sympathetic preganglionic neurons of the spinal cord receive excitatory inputs from neurones located in the hypothalamus and the brain stem, and send their axons to postganglionic sympathetic neurones of the paravertebral ganglia innervating the heart (FIGURE 1) (15). There is evidence that some sympathetic efferent postganglionic neurones may be located within the intrinsic cardiac ganglia, along with the parasympathetic postganglionic neurones (FIGURE 1) (reviewed in Ref. 1).

The sensory innervation of the heart is provided by afferent neurones located in the nodose and the dorsal root ganglia. These neurones are responsible for sending information to the CNS, including brain stem areas involved in autonomic control, and their activation can trigger reflex changes in sympathetic and parasympathetic efferent outflows to the heart. Sensory neurones innervating the heart also have been identified in intrathoracic extracardiac ganglia and intrinsic cardiac ganglia (1) (FIGURE 1). These sensory neurones are in a position to initiate local reflexes and, therefore, have a significant impact on heart rate and contractility via the modulation of the activity of efferent parasympathetic neurones located in the intrinsic cardiac ganglia (1).

Under resting conditions, the heart receives tonic influences from both sympathetic and parasympathetic efferent cardiac nerves, and it is generally believed that the control of the sympathetic and parasympathetic outflows to the heart is reciprocal in nature. The prevailing concept (conveyed by the physiology textbooks) of an autonomic balance postulates that activation of sympathetic or parasympathetic efferent limb is normally accompanied by inhibition of the other limb. This is certainly true for the operation of the baroreceptor reflex; however, there is evidence that some physiological responses may involve parallel co-activation of both sympathetic and parasympathetic drives to the heart (reviewed in Ref. 55). Compared with vagal control, cardiac sympathetic innervation appeared relatively late in evolution [it is not present in elasmobranch fish (74)], and concomitant activation of both autonomic limbs may be important to increase the “operational range” of the heart to meet metabolic demands of the body in ever-changing behavioral and environmental conditions.

## Myocardial Ischemia and Reperfusion

### Effect on Cardiomyocytes

Myocardial ischemia leads to metabolic and functional changes, and its duration determines the size of the myocardial infarction (28). Restitution of blood supply to an ischemic myocardial area is, therefore, an absolute prerequisite for tissue survival, and the longer the duration of ischemia the less myocardial tissue is likely to survive following reperfusion (21, 65). The size of the infarct and its temporal evolution are also determined by the area of myocardial ischemia (perfusion territory of the coronary artery distal to the site of its occlusion), i.e., area at risk, the level of collateral blood flow (46, 62), and multiple other covariates (e.g., systemic hemodynamics) (25). In small rodent hearts with low collateral blood flow, relatively large infarct size (>40% of the area at risk) is reached within 45–60 min of ischemia. In pigs, the time of infarct development is similar to that in rats. In dogs, collateral blood flow is relatively high, and infarction reaches ∼40% of the area at risk after 40 min of complete coronary occlusion and continues to develop over the next several hours (63). The time course of a first-time acute myocardial infarct evolution in humans appears to be significantly slower than in the most commonly used experimental animal models (including dogs) (22).

Treatment of acute ischemic damage has entered a new era, where patients' morbidity and mortality have been dramatically reduced by procedures ensuring a rapid return of blood flow to the ischemic myocardium (i.e., reperfusion therapy). The importance of prompt reperfusion is defined by the axiom, “time is muscle and muscle is life” (69). However, restoration of blood flow also results in a cascade of harmful events that leads to so-called myocardial reperfusion injury, which involves myocardial cell death, defined as the death of the cardiomyocytes that are still viable at the end of the period of ischemia.

### Effect on Cardiac Nervous System and Cardiac Reflexes

Major autonomic and sensory (afferent) innervations of the heart receive their own rich blood supply arising from extracardiac sources (27). However, the activity patterns of the sensory nerves innervating the myocardium can be dramatically affected by ions and active molecules (e.g., purines, peptides, hydroxyl radical, lactate, and others) released in response to tissue ischemia and/or reperfusion (1, 2). For example, during ischemia in an anaesthetized rat model, adenosine and its breakdown products progressively accumulate in the myocardial interstitium and undergo rapid washout at the onset of reperfusion (39).

Activation of cardiac afferents during ischemia/reperfusion leads to reflex changes in parasympathetic and sympathetic outflows to the heart. Observations in dogs revealed that “sympathetic” afferents are mainly distributed throughout the anterior and inferoposterior walls of the left ventricle and that their activation during ischemia in both locations leads to an increase in sympathetic drive, as evidenced by increases in renal sympathetic nerve discharge (49). In another study, simultaneous recordings from two thoracic sympathetic cardiac nerves revealed differential changes in the activity patterns of fibers supplying ischemic (decreased activity) and nonischemic (no change or increased activity) myocardium (53). Sympathoexcitation, which accompanies myocardial ischemia, appears to be largely independent of baroreceptor reflex operation (reviewed in Ref. 47).

Human data support the view that excitation of ventricular afferents is the predominant cause of general sympathetic activation, with similar increases in plasma norepinephrine observed in patients developing either anterior or inferior myocardial infarctions (32). In uncomplicated myocardial infarction, plasma norepinephrine concentration increases by approximately fivefold and is unlikely to have an adverse effect on myocardial function (67). However, reflex increases in cardiac sympathetic nerve activity combined with ischemia-induced reversed uptake may result in myocardial extracellular norepinephrine reaching >100 times its normal plasma concentration within 30 min of ischemia (67).

Afferent fibers that when activated trigger vagally mediated responses appear to be located primarily in the inferoposterior walls of the left ventricle (26). Vagal effects are observed early after the onset of myocardial ischemia occurring within the posterior wall of the left ventricle, whereas sympathetic activation occurs with some delay after an onset of an acute myocardial infarction within the anterior wall (44, 54). These reports indicate that the distinct patterns of reflex sympathetic and parasympathetic responses triggered by myocardial ischemia are dependent on the area of the left ventricle affected and may have a significant impact on cardiac function and coronary blood flow.

## Modulation of Myocardial Ischemia/Reperfusion Injury by Neural Mechanisms

Norepinephrine (in small amounts) and acetylcholine released within the myocardium from sympathetic and parasympathetic efferent terminals may protect the myocytes against lethal ischemia/reperfusion injury. Stimulation of α-adrenoceptors under certain circumstances can result in cardioprotection. Intracoronary administration of α_1a_-adrenoceptor agonist methoxamine limits the infarct size in dogs subjected to regional ischemia and reperfusion (36). Stimulation of α_1b_-adrenoceptors with phenylephrine was also shown to reduce infarct size in a rabbit model (75).

Potent cardioprotection could be established by a prototypical effector molecule of the parasympathetic nervous system: acetylcholine (ACh). In several studies, ACh was shown to be as potent as adenosine in protecting the myocardium against lethal ischemia/reperfusion injury (7, 59, 64, 80). Cardioprotection induced by ACh appears to be mediated via activation of muscarinic receptors and subsequent modulation of the activity of ATP-dependent potassium channels (7, 59, 80).

A number of studies have determined the extent of myocardial ischemia/reperfusion injury in conditions when neural control of the heart is experimentally altered. Electrical stimulation of the vagus nerve was found to be highly effective in limiting myocardial ischemia/reperfusion injury (6, 33). In rats, dogs, and cats, stimulation of the vagus nerve was also found to prevent ventricular arrhythmias during myocardial ischemia associated with enhanced cardiac sympathetic activity (9, 34, 50, 52, 82). In a recent study, genetic targeting and highly selective stimulation of ∼300–400 vagal preganglionic neurones located in the dorsal motor nucleus of the vagus nerve (DVMN) resulted in a dramatic ∼50% reduction of myocardial ischemia/reperfusion injury in a rat model (48). The beneficial effect of vagus nerve or DVMN stimulation during an acute myocardial infarction is independent of heart rate modulation (33, 48) and is abolished by blockade of muscarinic receptors with atropine (48). Anti-fibrillatory effect of vagus nerve stimulation may also recruit a mechanism mediated by nitric oxide (5).

Experimental studies in dogs demonstrated that spinal cord stimulation (SCS) reduces the baseline activity of intrinsic sensory cardiac neurones and suppresses their activation triggered by ventricular ischemia and reperfusion (13). In a rabbit model of ischemia/reperfusion injury, SCS (dorsal C8–T2 segments) was found to reduce infarct size when initiated before, but not after, ischemia onset (71). Cardioprotection induced by SCS was abolished by α_1_-adrenoceptor blockade with prazosin and significantly reduced by β-adrenoceptor blockade with timolol (71). A more recent study using the same experimental model demonstrated significant cardioprotection elicited by SCS applied at the cervical level (C1–C2) (70). Cardioprotection induced by cervical cord stimulation was abolished by section of the cord dorsally at C6 level as well as by either muscarinic (atropine), β-adrenoceptor (atenolol), or α-adrenoceptor (prazosin, yohimbine) blockade (70).

## Innate Mechanisms of Cardioprotection

Paradoxically, if the myocardium is exposed to short, nonlethal ischemia-reperfusion episodes, it becomes “preconditioned.” After such ischemic preconditioning (IPc), the myocardium becomes more tolerant to subsequent, much longer periods of ischemia. In a landmark study using anesthetized dogs, Murry et al. (51) demonstrated that four 5-min periods of left anterior descending coronary (LAD) artery occlusion, interspersed with 5-min reperfusion periods, resulted in profound reduction of the infarct size due to a subsequent 40-min-long occlusion of the same artery. IPc phenomenon was reproduced in a number of different experimental protocols in various species and with several endpoints of protection establishing IPc as a “gold standard” of cardioprotection.

Further experimental work has suggested that protective effects of IPc occur due to modulation of reperfusion injury, and consequently the idea suggesting that the preconditioning stimulus could only be effective when applied before ischemia was challenged (56). In the experiments using dogs, Zhao et al. (81) demonstrated a reduction in infarct size following three 30-s periods of LAD artery occlusion applied at the onset of reperfusion, which followed a 60-min occlusion period. The term “ischemic postconditioning” (IPost) was proposed to describe this protective phenomenon, and that study triggered significant interest in the opportunity of effectively targeting reperfusion injury if protective mechanisms of IPc and IPost are identified. However, widespread clinical use of IPc is difficult to implement due to unpredicted onset of the ischemic event and technical limitations requiring complex invasive procedures and application of direct myocardial ischemia.

Importantly, the myocardium can also be protected by remote ischemic preconditioning (RPc), the phenomenon in which cycles of brief ischemia and reperfusion of an organ or tissue protect a remote organ or tissue against ischemia/reperfusion injury. Przyklenk et al. (58) first demonstrated that brief ischemia of the circumflex artery reduced infarct size in the cardiac tissue supplied by the LAD artery. Subsequent studies have shown that cardioprotection can also be established by application of an ischemic stimulus to various organs distant to the heart (e.g., limbs and kidney). RPc results in the levels of protection comparable to that achieved by “classical” local IPc (42, 76) and at the level of the cardiomyocyte may recruit distinct or similar signaling pathways (19, 23, 38, 76, 78). Remarkably, myocardial ischemia/reperfusion injury can also be reduced by remote ischemic postconditioning, which confers even stronger cardioprotection than the IPost applied directly to the myocardium (17).

There is an ongoing debate on the mechanisms of how a “preconditioning stimulus” is relayed from the remote ischemic tissue/organ to the heart. Relay pathways of RPc-induced cardioprotection were suggested to involve humoral factor(s) produced during ischemia/reperfusion of the remote tissue and released into the systemic circulation (20, 35, 37, 43, 68, 76), or a neural component (3, 11, 14, 31, 43, 45, 48, 73), or both (43, 61).

## Neural Mechanisms of Remote Ischemic Preconditioning Cardioprotection

Gho et al. (14) demonstrated that the ganglion blocker hexamethonium abolishes cardioprotection established by RPc, providing the first evidence that neural (autonomic) pathways mediate this phenomenon. Subsequent studies reported that adenosine, bradykinin, and calcitonin gene-related peptide are all important components in the neural mechanisms underlying RPc (66, 77). The exact neural pathways remained unclear but appeared to be also important for human remote preconditioning, as demonstrated using the model of forearm ischemic endothelial injury assessed by flow-mediated dilation (45). On the other hand, an experimental study in pigs demonstrated that RPc (induced by transient limb ischemia) leads to a significant protection of the transplanted (i.e., completely denervated) heart (37). These data suggest that, in addition to a neural mechanism, a circulating substance (or a group of substances) may contribute to cardioprotective effects of RPc.

Indeed, plasma dialysate obtained from patients receiving RPc stimulus protects the rabbit heart (Langendorff preparation) against ischemia/reperfusion injury (29). Interestingly, plasma dialysates obtained from diabetic patients with peripheral neuropathy were not effective (29), supporting the idea that the release mechanism of preconditioning humoral factor(s) involves recruitment of a neural signaling pathway(s). Similar conclusions were also reached by Redington et al. (61), who demonstrated significant cardioprotection following application of plasma dialysates obtained from the experimental animals receiving RPc stimulus or following activation of the peripheral sensory fibers. Taken together, these data suggest that cardioprotection induced by RPc is dependent on particular experimental conditions and appears to be species and model dependent.

In an attempt to identify humoral factor(s) that mediate remote ischemic preconditioning, Lang et al. (40) performed proteomic analysis of the blood samples obtained from experimental animals subjected to myocardial ischemia/reperfusion injury with or without application of the RPc stimulus. The study failed to detect differential expression of proteins that possess a known signaling function and did not support the existence of a humoral mediator of RPc with a molecular weight of >8 kDa (40). In contrast, a more recent study conducted in healthy adult volunteers reported that RPc evokes differential regulation (up- or downregulation) of various plasma proteins (24). Increases in the number of upregulated peptides was cumulative with each cycle of the arm ischemia/reperfusion. The proteins identified are linked to the control of the acute phase response and regulation of various cellular functions, suggesting that the humoral mechanisms that mediate RPc cardioprotection may involve complex interaction of multiple redundant signaling pathways.

The results of our studies using a rat model of myocardial ischemia/reperfusion injury confirmed that RPc stimulus applied to the limbs (induced by 15 min of occlusion of femoral arteries) significantly reduces myocardial ischemia/reperfusion injury (3). It was found that bilateral cervical vagotomy, surgical denervation of the ischemic limbs by sectioning the sciatic and femoral nerves, or permanent functional depletion of sensory nerves induced by neonatal systemic capsaicin (neurotoxin, the active component of chilli peppers) treatment effectively abolishes RPc cardioprotection (3, 16).

Interestingly, strong cardioprotection is elicited when capsaicin is applied topically to stimulate sensory neurones innervating remote organ/tissue (3, 31, 61). The same population of sensory fibers (nociceptors) are likely to be activated by trauma associated with surgical incision, which was found to induce potent cardioprotection of the mouse and canine hearts (18, 31). The type of sensory fibers, activation of which in response to tissue ischemia/reperfusion or trauma mediates cardioprotection, can be defined as “capsaicin-sensitive” afferents, C- and Aδ-fibers, which are excited (and at high doses destroyed) by capsaicin.

**FIGURE 2.**
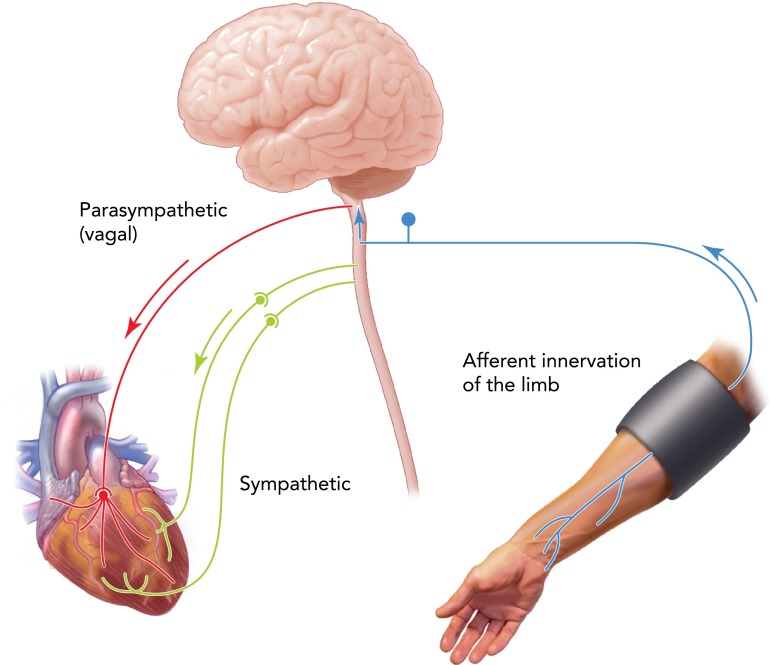
Diagrammatic representation of a remote preconditioning reflex Remote ischemic preconditioning cardioprotection is hypothesized to be mediated via activation of a neural pathway comprising C-fiber sensory innervation of the remote ischemic organ/tissue and vagal innervation of the heart (16). Remote preconditioning of trauma may also involve sympathetic recruitment (31).

Failure of remote ischemia/reperfusion to establish cardioprotection in conditions of bilateral cervical vagotomy or systemic muscarinic receptor blockade with atropine (3, 10, 16, 48) suggests an important role of the parasympathetic nervous system in mediating the RPc phenomenon. This view is supported by human data showing that intermittent arm ischemia increases parasympathetic tone (12). This increase would be expected to have a beneficial effect on the heart in view of strong experimental evidence (reviewed above) demonstrating potent cardioprotection induced by vagus nerve stimulation (6, 33) or direct stimulation of vagal preganglionic neurones (48). A concept of a “remote preconditioning reflex” that involves sensory input from the remote ischemic organ/tissue and protects the heart via increased cardiac parasympathetic activity was recently proposed (FIGURE 2) (15).

A recent study attempted to disentangle humoral and neural mechanisms of RPc cardioprotection by targeted inhibition of a discrete central nervous substrate that generates parasympathetic tone. Cell-specific silencing of vagal preganglionic neurones located in the brain stem dorsal motor nucleus of the vagus nerve (DVMN) was achieved by transducing them with a viral vector to express an inhibitory *Drosophila* allatostatin receptor (48). RPc cardioprotection was completely abolished when transduced DVMN neurones were silenced by application of the selective ligand insect peptide allatostatin (48). These data revealed a crucial dependence of interorgan protection against ischemia/reperfusion injury upon the activity of a distinct population of vagal preganglionic neurones. DVMN neurones are believed to contribute to tonic C-fiber-mediated parasympathetic innervation of the heart (30), and that study also confirmed abundant cholinergic innervation of the rat left ventricle (48). These results do not exclude the involvement of humoral factor(s) but demonstrate that to establish cardioprotection a “humoral pathway” of RPc requires a certain level of activity of the DVMN vagal preganglionic neurones.

In conclusion, in this account, we have attempted to highlight the importance of neural mechanisms in protecting the heart against lethal myocardial ischemia/reperfusion injury. A large body of evidence demonstrates that the extent of myocardial infarction can be significantly reduced by experimentally induced changes in the activities of parasympathetic and sympathetic efferent fibers innervating the heart. Myocardial ischemia/reperfusion (via activation of cardiac afferents) and ischemia/reperfusion of tissues located remote from the heart (via activation of C-fiber afferents) can trigger distinct patterns of parasympathetic and sympathetic responses. The strength of these responses may be sufficient to establish cardioprotection, and it is conceivable that activation of these reflex pathways contribute to powerful innate mechanisms of cardioprotection underlying the (pre)conditioning phenomena. Promising results of clinical trials in patients with acute myocardial infarction (4) may lead to widespread introduction of remote ischemic conditioning procedure(s) into clinical practice. However, there are also negative reports (57, 60). Parasympathetic tone decreases with age and could be severely diminished or even absent in many disease states, perhaps rendering many patents insensitive to this procedure. We suggest that effective heart conditioning strategies should be capable of increasing cardiac parasympathetic activity and by doing so should be highly effective in reducing myocardial injury and decreasing morbidity and mortality of patients with ischemic heart disease.

## References

[1] Armour JA Myocardial ischaemia and the cardiac nervous system. Cardiovasc Res 41: 41–54, 1999 10325952 10.1016/s0008-6363(98)00252-1

[2] Armour JA Cardiac neuronal hierarchy in health and disease. Am J Physiol Regul Integr Comp Physiol 287: R262–R271, 2004 15271675 10.1152/ajpregu.00183.2004

[3] Basalay M Barsukevich V Mastitskaya S Mrochek A Pernow J Sjoquist PO Ackland GL Gourine AV Gourine A Remote ischemic pre- and delayed postconditioning - similar degree of cardioprotection but distinct mechanisms. Exp Physiol 97: 908–917, 2012 22427438 10.1113/expphysiol.2012.064923 PMC3470925

[4] Botker HE Kharbanda R Schmidt MR Bottcher M Kaltoft AK Terkelsen CJ Munk K Andersen NH Hansen TM Trautner S Lassen JF Christiansen EH Krusell LR Kristensen SD Thuesen L Nielsen SS Rehling M Sorensen HT Redington AN Nielsen TT Remote ischemic conditioning before hospital admission, as a complement to angioplasty, and effect on myocardial salvage in patients with acute myocardial infarction: a randomised trial. Lancet 375: 727–734, 2010 20189026 10.1016/S0140-6736(09)62001-8

[5] Brack KE Coote JH Ng GA Vagus nerve stimulation protects against ventricular fibrillation independent of muscarinic receptor activation. Cardiovasc Res 91: 437–446, 2011 21576131 10.1093/cvr/cvr105

[6] Calvillo L Vanoli E Andreoli E Besana A Omodeo E Gnecchi M Zerbi P Vago G Busca G Schwartz PJ Vagal stimulation, through its nicotinic action, limits infarct size and the inflammatory response to myocardial ischemia and reperfusion. J Cardiovasc Pharmacol 58: 500–507, 2011 21765369 10.1097/FJC.0b013e31822b7204

[7] Cohen MV Yang XM Liu GS Heusch G Downey JM Acetylcholine, bradykinin, opioids, and phenylephrine, but not adenosine, trigger preconditioning by generating free radicals and opening mitochondrial K(ATP) channels. Circ Res 89: 273–278, 2001 11485978 10.1161/hh1501.094266

[8] Coote JH Myths and realities of the cardiac vagus. J Physiol. In press 10.1113/jphysiol.2013.257758 PMC3779103 23878363

[9] Corr PB Gillis RA Role of the vagus nerves in the cardiovascular changes induced by coronary occlusion. Circulation 49: 86–97, 1974 4808850 10.1161/01.cir.49.1.86

[10] Donato M Buchholz B Rodriguez M Perez V Inserte J Garcia-Dorado D Gelpi RJ Role of the parasympathetic nervous system in cardioprotection by remote hindlimb ischemic preconditioning. Exp Physiol 98: 425–434, 2013 22872660 10.1113/expphysiol.2012.066217

[11] Dong JH Liu YX Ji ES He RR [Limb ischemic preconditioning reduces infarct size following myocardial ischemia-reperfusion in rats]. Sheng Li Xue Bao 56: 41–46, 2004 14985828

[12] Enko K Nakamura K Yunoki K Miyoshi T Akagi S Yoshida M Toh N Sangawa M Nishii N Nagase S Kohno K Morita H Kusano KF Ito H Intermittent arm ischemia induces vasodilatation of the contralateral upper limb. J Physiol Sci 61: 507–513, 2011 21901641 10.1007/s12576-011-0172-9 PMC10718035

[13] Foreman RD Linderoth B Ardell JL Barron KW Chandler MJ Hull SS Jr TerHorst GJ DeJongste MJ Armour JA Modulation of intrinsic cardiac neurons by spinal cord stimulation: implications for its therapeutic use in angina pectoris. Cardiovasc Res 47: 367–375, 2000 10946073 10.1016/s0008-6363(00)00095-x

[14] Gho BC Schoemaker RG van den Doel MA Duncker DJ Verdouw PD Myocardial protection by brief ischemia in noncardiac tissue. Circulation 94: 2193–2200, 1996 8901671 10.1161/01.cir.94.9.2193

[15] Gilbey MP Sympathetic rhythms and nervous integration. Clin Exp Pharmacol Physiol 34: 356–361, 2007 17324150 10.1111/j.1440-1681.2007.04587.x

[16] Gourine A Gourine AV Mastitskaya S Ackland G “Remote preconditioning reflex”: a neural pathway of cardioprotection during myocardial ischaemia and reperfusion induced by remote ischemic preconditioning. Eur Heart J 31: 319, 2010

[17] Gritsopoulos G Iliodromitis EK Zoga A Farmakis D Demerouti E Papalois A Paraskevaidis IA Kremastinos DT Remote postconditioning is more potent than classic postconditioning in reducing the infarct size in anesthetized rabbits. Cardiovasc Drugs Ther 23: 193–198, 2009 19255833 10.1007/s10557-009-6168-5

[18] Gross GJ Baker JE Moore J Falck JR Nithipatikom K Abdominal surgical incision induces remote preconditioning of trauma (RPCT) via activation of bradykinin receptors (BK2R) and the cytochrome P450 epoxygenase pathway in canine hearts. Cardiovasc Drugs Ther 25: 517–522, 2011 21786213 10.1007/s10557-011-6321-9 PMC3329256

[19] Hausenloy DJ Iliodromitis EK Andreadou I Papalois A Gritsopoulos G Anastasiou-Nana M Kremastinos DT Yellon DM Investigating the signal transduction pathways underlying remote ischemic conditioning in the porcine heart. Cardiovasc Drugs Ther 26: 87–93, 2012 22207395 10.1007/s10557-011-6364-y

[20] Hausenloy DJ Yellon DM Remote ischemic preconditioning: underlying mechanisms and clinical application. Cardiovasc Res 79: 377–386, 2008 18456674 10.1093/cvr/cvn114

[21] Hearse DJ Myocardial protection during ischemia and reperfusion. Mol Cell Biochem 186: 177–184, 1998 9774199

[22] Hedstrom E Engblom H Frogner F Astrom-Olsson K Ohlin H Jovinge S Arheden H Infarct evolution in man studied in patients with first-time coronary occlusion in comparison to different species: implications for assessment of myocardial salvage. J Cardiovasc Magn Reson 11: 38, 2009 19775428 10.1186/1532-429X-11-38 PMC2758877

[23] Heinen NM Putz VE Gorgens JI Huhn R Gruber Y Barthuber C Preckel B Pannen BH Bauer I Cardioprotection by remote ischemic preconditioning exhibits a signaling pattern different from local ischemic preconditioning. Shock 36: 45–53, 2011 21478813 10.1097/SHK.0b013e31821d8e77

[24] Hepponstall M Ignjatovic V Binos S Monagle P Jones B Cheung MH d'Udekem Y Konstantinov IE Remote ischemic preconditioning (RIPC) modifies plasma proteome in humans. PLos One 7: e48284, 2012 23139772 10.1371/journal.pone.0048284 PMC3489679

[25] Heusch G The regional myocardial flow-function relationship: a framework for an understanding of acute ischemia, hibernation, stunning and coronary microembolization. 1980. Circ Res 112: 1535–1537, 2013 23743225 10.1161/CIRCRESAHA.113.301446

[26] Inoue H Zipes DP Increased afferent vagal responses produced by epicardial application of nicotine on the canine posterior left ventricle. Am Heart J 114: 757–764, 1987 3661366 10.1016/0002-8703(87)90786-1

[27] Janes RD Johnstone DE Armour JA Functional integrity of intrinsic cardiac nerves located over an acute transmural myocardial infarction. Can J Physiol Pharmacol 65: 64–69, 1987 3567722 10.1139/y87-012

[28] Jennings RB Murry CE Steenbergen C Jr Reimer KA Development of cell injury in sustained acute ischemia. Circulation 82: 2–12, 1990 2394018

[29] Jensen RV Stottrup NB Kristiansen SB Botker HE Release of a humoral circulating cardioprotective factor by remote ischemic preconditioning is dependent on preserved neural pathways in diabetic patients. Basic Res Cardiol 107: 285, 2012 22821347 10.1007/s00395-012-0285-1

[30] Jones JF Vagal control of the rat heart. Exp Physiol 86: 797–801, 2001 11698976 10.1111/j.1469-445x.2001.tb00047.x

[31] Jones WK Fan GC Liao S Zhang JM Wang Y Weintraub NL Kranias EG Schultz JE Lorenz J Ren X Peripheral nociception associated with surgical incision elicits remote nonischemic cardioprotection via neurogenic activation of protein kinase C signaling. Circulation 120: 1–9, 2009 10.1161/CIRCULATIONAHA.108.843938 PMC2845316 19752352

[32] Karlsberg RP Cryer PE Roberts R Serial plasma catecholamine response early in the course of clinical acute myocardial infarction: relationship to infarct extent and mortality. Am Heart J 102: 24–29, 1981 7246410 10.1016/0002-8703(81)90408-7

[33] Katare RG Ando M Kakinuma Y Arikawa M Handa T Yamasaki F Sato T Vagal nerve stimulation prevents reperfusion injury through inhibition of opening of mitochondrial permeability transition pore independent of the bradycardiac effect. J Thorac Cardiovasc Surg 137: 223–231, 2009 19154929 10.1016/j.jtcvs.2008.08.020

[34] Kent KM Smith ER Redwood DR Epstein SE Electrical stability of acutely ischemic myocardium. Influences of heart rate and vagal stimulation. Circulation 47: 291–298, 1973 4684930 10.1161/01.cir.47.2.291

[35] Kingma JG Jr Simard D Voisine P Rouleau JR Role of the autonomic nervous system in cardioprotection by remote preconditioning in isoflurane-anaesthetized dogs. Cardiovasc Res 89: 384–391, 2011 20876586 10.1093/cvr/cvq306

[36] Kitakaze M Hori M Kamada T Role of adenosine and its interaction with alpha adrenoceptor activity in ischemic and reperfusion injury of the myocardium. Cardiovasc Res 27: 18–27, 1993 8384527 10.1093/cvr/27.1.18

[37] Konstantinov IE Li J Cheung MM Shimizu M Stokoe J Kharbanda RK Redington AN Remote ischemic preconditioning of the recipient reduces myocardial ischemia-reperfusion injury of the denervated donor heart via a K_ATP_ channel-dependent mechanism. Transplantation 79: 1691–1695, 2005 15973170 10.1097/01.tp.0000159137.76400.5d

[38] Kristiansen SB Henning O Kharbanda RK Nielsen-Kudsk JE Schmidt MR Redington AN Nielsen TT Botker HE Remote preconditioning reduces ischemic injury in the explanted heart by a K_ATP_ channel-dependent mechanism. Am J Physiol Heart Circ Physiol 288: H1252–H1256, 2005 15498829 10.1152/ajpheart.00207.2004

[39] Kuzmin AI Gourine AV Molosh AI Lakomkin VL Vassort G Effects of preconditioning on myocardial interstitial levels of ATP and its catabolites during regional ischemia and reperfusion in the rat. Basic Res Cardiol 95: 127–136, 2000 10826505 10.1007/s003950050174

[40] Lang SC Elsasser A Scheler C Vetter S Tiefenbacher CP Kubler W Katus HA Vogt AM Myocardial preconditioning and remote renal preconditioning-identifying a protective factor using proteomic methods? Basic Res Cardiol 101: 149–158, 2006 16283592 10.1007/s00395-005-0565-0

[41] Lewis ME Al-Khalidi AH Bonser RS Clutton-Brock T Morton D Paterson D Townend JN Coote JH Vagus nerve stimulation decreases left ventricular contractility in vivo in the human and pig heart. J Physiol 534: 547–552, 2001 11454971 10.1111/j.1469-7793.2001.00547.x PMC2278718

[42] Liem DA te Lintel HM Manintveld OC Boomsma F Verdouw PD Duncker DJ Myocardium tolerant to an adenosine-dependent ischemic preconditioning stimulus can still be protected by stimuli that employ alternative signaling pathways. Am J Physiol Heart Circ Physiol 288: H1165–H1172, 2005 15486028 10.1152/ajpheart.00899.2004

[43] Lim SY Yellon DM Hausenloy DJ The neural and humoral pathways in remote limb ischemic preconditioning. Basic Res Cardiol 105: 651–655, 2010 20449597 10.1007/s00395-010-0099-y

[44] Lombardi F Sandrone G Spinnler MT Torzillo D Lavezzaro GC Brusca A Malliani A Heart rate variability in the early hours of an acute myocardial infarction. Am J Cardiol 77: 1037–1044, 1996 8644654 10.1016/s0002-9149(96)00127-0

[45] Loukogeorgakis SP Panagiotidou AT Broadhead MW Donald A Deanfield JE MacAllister RJ Remote ischemic preconditioning provides early and late protection against endothelial ischemia-reperfusion injury in humans: role of the autonomic nervous system. J Am Coll Cardiol 46: 450–456, 2005 16053957 10.1016/j.jacc.2005.04.044

[46] Lowe JE Reimer KA Jennings RB Experimental infarct size as a function of the amount of myocardium at risk. Am J Pathol 90: 363–379, 1978 623206 PMC2018154

[47] Malliani A Montano N Emerging excitatory role of cardiovascular sympathetic afferents in pathophysiological conditions. Hypertension 39: 63–68, 2002 11799080 10.1161/hy0102.099200

[48] Mastitskaya S Marina N Gourine A Gilbey MP Spyer KM Teschemacher AG Kasparov S Trapp S Ackland GL Gourine AV Cardioprotection evoked by remote ischemic preconditioning is critically dependent on the activity of vagal pre-ganglionic neurones. Cardiovasc Res 95: 487–494, 2012 22739118 10.1093/cvr/cvs212 PMC3422080

[49] Minisi AJ Thames MD Distribution of left ventricular sympathetic afferents demonstrated by reflex responses to transmural myocardial ischemia and to intracoronary and epicardial bradykinin. Circulation 87: 240–246, 1993 8419013 10.1161/01.cir.87.1.240

[50] Mioni C Bazzani C Giuliani D Altavilla D Leone S Ferrari A Minutoli L Bitto A Marini H Zaffe D Botticelli AR Iannone A Tomasi A Bigiani A Bertolini A Squadrito F Guarini S Activation of an efferent cholinergic pathway produces strong protection against myocardial ischemia/reperfusion injury in rats. Crit Care Med 33: 2621–2628, 2005 16276189 10.1097/01.ccm.0000186762.05301.13

[51] Murry CE Jennings RB Reimer KA Preconditioning with ischemia: a delay of lethal cell injury in ischemic myocardium. Circulation 74: 1124–1136, 1986 3769170 10.1161/01.cir.74.5.1124

[52] Myers RW Pearlman AS Hyman RM Goldstein RA Kent KM Goldstein RE Epstein SE Beneficial effects of vagal stimulation and bradycardia during experimental acute myocardial ischemia. Circulation 49: 943–947, 1974 4828616 10.1161/01.cir.49.5.943

[53] Neely BH Hageman GR Differential cardiac sympathetic activity during acute myocardial ischemia. Am J Physiol Heart Circ Physiol 258: H1534–H1541, 1990 10.1152/ajpheart.1990.258.5.H1534 2337185

[54] Pantridge JF Webb SW Adgey AA Arrhythmias in the first hours of acute myocardial infarction. Prog Cardiovasc Dis 23: 265–278, 1981 7008079 10.1016/0033-0620(81)90016-5

[55] Paton JF Boscan P Pickering AE Nalivaiko E The yin and yang of cardiac autonomic control: vago-sympathetic interactions revisited. Brain Res Rev 49: 555–565, 2005 16269319 10.1016/j.brainresrev.2005.02.005

[56] Piper HM Garcia-Dorado D Ovize M A fresh look at reperfusion injury. Cardiovasc Res 38: 291–300, 1998 9709390 10.1016/s0008-6363(98)00033-9

[57] Prasad A Gossl M Hoyt J Lennon RJ Polk L Simari R Holmes DR Jr Rihal CS Lerman A Remote ischemic preconditioning immediately before percutaneous coronary intervention does not impact myocardial necrosis, inflammatory response, and circulating endothelial progenitor cell counts: a single center randomized sham controlled trial. Catheter Cardiovasc Interv 81: 930–936, 2013 22517646 10.1002/ccd.24443

[58] Przyklenk K Bauer B Ovize M Kloner RA Whittaker P Regional ischemic ‘preconditioning’ protects remote virgin myocardium from subsequent sustained coronary occlusion. Circulation 87: 893–899, 1993 7680290 10.1161/01.cir.87.3.893

[59] Qian YZ Levasseur JE Yoshida K Kukreja RC KATP channels in rat heart: blockade of ischemic and acetylcholine-mediated preconditioning by glibenclamide. Am J Physiol Heart Circ Physiol 271: H23–H28, 1996 10.1152/ajpheart.1996.271.1.H23 8760153

[60] Rahman IA Mascaro JG Steeds RP Frenneaux MP Nightingale P Gosling P Townsend P Townend JN Green D Bonser RS Remote ischemic preconditioning in human coronary artery bypass surgery: from promise to disappointment? Circulation 122: 53–59, 2010 10.1161/CIRCULATIONAHA.109.926667 20837926

[61] Redington KL Disenhouse T Strantzas SC Gladstone R Wei C Tropak MB Dai X Manlhiot C Li J Redington AN Remote cardioprotection by direct peripheral nerve stimulation and topical capsaicin is mediated by circulating humoral factors. Basic Res Cardiol 107: 1–10, 2012 10.1007/s00395-011-0241-5 22231674

[62] Reimer KA Jennings RB The “wavefront phenomenon” of myocardial ischemic cell death. II. Transmural progression of necrosis within the framework of ischemic bed size (myocardium at risk) and collateral flow. Lab Invest 40: 633–644, 1979 449273

[63] Reimer KA Lowe JE Rasmussen MM Jennings RB The wavefront phenomenon of ischemic cell death. I. Myocardial infarct size vs duration of coronary occlusion in dogs. Circulation 56: 786–794, 1977 912839 10.1161/01.cir.56.5.786

[64] Richard V Blanc T Kaeffer N Tron C Thuillez C Myocardial and coronary endothelial protective effects of acetylcholine after myocardial ischaemia and reperfusion in rats: role of nitric oxide. Br J Pharmacol 115: 1532–1538, 1995 8564215 10.1111/j.1476-5381.1995.tb16647.x PMC1908894

[65] Sanada S Komuro I Kitakaze M Pathophysiology of myocardial reperfusion injury: preconditioning, postconditioning, and translational aspects of protective measures. Am J Physiol Heart Circ Physiol 301: H1723–H1741, 2011 21856909 10.1152/ajpheart.00553.2011

[66] Schoemaker RG van Heijningen CL Bradykinin mediates cardiac preconditioning at a distance. Am J Physiol Heart Circ Physiol 278: H1571–H1576, 2000 10775135 10.1152/ajpheart.2000.278.5.H1571

[67] Schomig A Catecholamines in myocardial ischemia. Systemic and cardiac release. Circulation 82: 13–22, 1990 2203558

[68] Shimizu M Tropak M Diaz RJ Suto F Surendra H Kuzmin E Li J Gross G Wilson GJ Callahan J Redington AN Transient limb ischaemia remotely preconditions through a humoral mechanism acting directly on the myocardium: evidence suggesting cross-species protection. Clin Sci (Lond) 117: 191–200, 2009 19175358 10.1042/CS20080523

[69] Simoons ML Boersma E Maas AC Deckers JW Management of myocardial infarction: the proper priorities. Eur Heart J 18: 896–899, 1997 9183578 10.1093/oxfordjournals.eurheartj.a015375

[70] Southerland EM Gibbons DD Smith SB Sipe A Williams CA Beaumont E Armour JA Foreman RD Ardell JL Activated cranial cervical cord neurons affect left ventricular infarct size and the potential for sudden cardiac death. Auton Neurosci 169: 34–42, 2012 22502863 10.1016/j.autneu.2012.03.003 PMC3361540

[71] Southerland EM Milhorn DM Foreman RD Linderoth B DeJongste MJ Armour JA Subramanian V Singh M Singh K Ardell JL Preemptive, but not reactive, spinal cord stimulation mitigates transient ischemia-induced myocardial infarction via cardiac adrenergic neurons. Am J Physiol Heart Circ Physiol 292: H311–H317, 2007 16920800 10.1152/ajpheart.00087.2006

[72] Spyer KM Annual review prize lecture. Central nervous mechanisms contributing to cardiovascular control. J Physiol 474: 1–19, 1994 8014887 10.1113/jphysiol.1994.sp019997 PMC1160290

[73] Steensrud T Li J Dai X Manlhiot C Kharbanda RK Tropak M Redington A Pretreatment with the nitric oxide donor SNAP or nerve transection blocks humoral preconditioning by remote limb ischemia or intra-arterial adenosine. Am J Physiol Heart Circ Physiol 299: H1598–H1603, 2010 20802131 10.1152/ajpheart.00396.2010

[74] Taylor EW Jordan D Coote JH Central control of the cardiovascular and respiratory systems and their interactions in vertebrates. Physiol Rev 79: 855–916, 1999 10390519 10.1152/physrev.1999.79.3.855

[75] Tsuchida A Liu Y Liu GS Cohen MV Downey JM Alpha 1-adrenergic agonists precondition rabbit ischemic myocardium independent of adenosine by direct activation of protein kinase C. Circ Res 75: 576–585, 1994 7914839 10.1161/01.res.75.3.576

[76] Weinbrenner C Nelles M Herzog N Sarvary L Strasser RH Remote preconditioning by infrarenal occlusion of the aorta protects the heart from infarction: a newly identified non-neuronal but PKC-dependent pathway. Cardiovasc Res 55: 590–601, 2002 12160957 10.1016/s0008-6363(02)00446-7

[77] Wolfrum S Nienstedt J Heidbreder M Schneider K Dominiak P Dendorfer A Calcitonin gene related peptide mediates cardioprotection by remote preconditioning. Regul Pept 127: 217–224, 2005 15680490 10.1016/j.regpep.2004.12.008

[78] Wolfrum S Schneider K Heidbreder M Nienstedt J Dominiak P Dendorfer A Remote preconditioning protects the heart by activating myocardial PKCepsilon-isoform. Cardiovasc Res 55: 583–589, 2002 12160956 10.1016/s0008-6363(02)00408-x

[79] Xenopoulos NP Applegate RJ The effect of vagal stimulation on left ventricular systolic and diastolic performance. Am J Physiol Heart Circ Physiol 266: H2167–H2173, 1994 10.1152/ajpheart.1994.266.6.H2167 8023978

[80] Yamaguchi F Nasa Y Yabe K Ohba S Hashizume Y Ohaku H Furuhama K Takeo S Activation of cardiac muscarinic receptor and ischemic preconditioning effects in in situ rat heart. Heart Vessels 12: 74–83, 1997 9403311 10.1007/BF02820870

[81] Zhao ZQ Corvera JS Halkos ME Kerendi F Wang NP Guyton RA Vinten-Johansen J Inhibition of myocardial injury by ischemic postconditioning during reperfusion: comparison with ischemic preconditioning. Am J Physiol Heart Circ Physiol 285: H579–H588, 2003 12860564 10.1152/ajpheart.01064.2002

[82] Zuanetti G De Ferrari GM Priori SG Schwartz PJ Protective effect of vagal stimulation on reperfusion arrhythmias in cats. Circ Res 61: 429–435, 1987 3621502 10.1161/01.res.61.3.429

